# Analysis of changes in the spatiotemporal characteristics of impervious surfaces and their influencing factors in the Central Plains Urban Agglomeration of China from 2000 to 2018

**DOI:** 10.1016/j.heliyon.2023.e18849

**Published:** 2023-08-01

**Authors:** Chunhong Zhao, Huabo Zhang, Haiying Wang, Jinyi Zhao

**Affiliations:** a The College of Geography and Environment Science, Henan University, Kaifeng, 475004, China; b Key Laboratory of Geospatial Technology for Middle and Lower Yellow River Regions, Ministry of Education, Kaifeng, 475004, China; c Institute of Urban Big Data, Henan University, Kaifeng, 475004, China

**Keywords:** Impervious surface area, Spatiotemporal feature change, GeoDetector, Central Plains Urban Agglomeration

## Abstract

The change of the impervious surface area (ISA) is an important feature of urban spatial expansion. Understanding the spatial and temporal change characteristics of urban impervious surfaces and their influencing factors is of great significance to the planning, management, and urbanization development of cities. This paper adopts a global artificial impervious surface dataset, with a resolution of 30 m, calculates and processes the data based on the ArcGIS platform, adopts the MK trend test method, introduces the dynamic degree, qualitatively and quantitatively analyses the changing characteristics of the ISA in the Central Plains Urban Agglomeration (CPUA), and analyzes the influencing factors of ISA changes using GeoDetector. The results show that the ISA dynamic degree was significantly enhanced from 2000 to 2018, which increased 1.86 times, indicating the accelerated outward expansion of the CPUA and the rapid level of urban development during that period. The explanatory power of the greenery coverage area on the change of the ISA in the CPUA during 2000–2018 was the strongest; the same factor has different explanatory powers for the ISA in different periods. The influencing factors have an enhanced relationship between two and two, including two-factor enhancement and nonlinear enhancement, in 2000–2009, during which the interaction level of the greenery coverage area and the GDP per capita was strong, while from 2009 to 2018, the interaction between transportation and other factors was significantly strong.

## 1 Introduction

With rapid social and economic development, the urbanization development rate in China has been booming, and as of 2018, the urbanization rate in China had grown to 59.2% [1]. The development of urbanization is generally manifested by an increase in the impervious surface area (ISA), which is a surface covered by various impervious construction materials such as tiles, asphalt, and cement concrete [2]. The ISA has a close relationship with the development of urbanization, and changes in the impervious surfaces can directly reflect the development and expansion of cities. With economicrapid development, in the process of urbanization, a large number of pervious surfaces such as vegetation and farmland are transformed into impervious surfaces. The increase of impervious surfaces will lead to changes in land cover, resulting in a series of environmental problems endangering human health, such as urban waterlogging, the urban heat island effect, climate change, water resource shortage and ecological environment deterioration [3,4]. Urban impervious surface area is a key indicator of the extent of urban development and the quality of urban ecosystems [5]. By studying the spatiotemporal characteristics of impervious surfaces and their intrinsic mechanisms and grasping the changing trends of regional impervious surfaces, it is important for researchers of urban planning, water resources management, environmental protection and disaster warning, to provide decision makers with corresponding valuable information and data support to promote sustainable regional development. As such, the study of spatial and temporal changes of urban impervious surfaces has received attention from government departments and scholars in many countries [6]. As a new growth pole in the central region of China, the Central Plains Urban Agglomeration (CPUA) is a bridge linking the development of central and western China, and it is of strategic significance to promote the development of the CPUA to accelerate the rise of the central region, promote the construction of new urbanization, and expand the new pattern of economic development in China. Studies of the changes of the spatial and temporal characteristics of the impervious surfaces of the CPUA and their influencing factors can effectively link its inherent urban development chronology and spatial characteristics with future development directions and trends, and provide an important reference for the future urban agglomeration development in China [7].

At present, widely used ISA datasets are GlobeLand30, NUACI, HBASE, GHSL, and FROM-GLC, which differ in terms of research time series, accuracy, resolution, etc. GlobeLand30 is a global 30 m land cover dataset generated based on the POK method, which has high resolution and is often used for remote sensing extraction accuracy verification; however, it has only one year of data, making it unsuitable for long time series analyses [8,9]. NUACI [10], a dynamic urban land expansion dataset, is a multi-temporal global 30 m impervious surface dataset developed based on spectral indices; it is based on Landsat imagery from 1990 to 2010 and extracts impervious surface data that have five year intervals. The HBASE dataset is the first global 30 m artificial impervious cover dataset derived from 2010 Global Land Survey (GLS) Landsat data, which was produced by combining instrument resolution training data, OSM, VIIRS NTL, GLS Landsat SR, and MODIS normalized vegetation index products. GHSL was derived using a global information baseline describing the spatial evolution of human settlements over the past 40 years, developed by machine learning models trained with high-resolution samples from 1975, 1990, 2000, and 2015 and multi-period Landsat imagery. Comparatively, FROM-GLC [11] is the latest global artificial impervious area (GAIA) dataset produced by Gong Peng's team at Tsinghua University, which has the longest time series (1985–2018), a spatial resolution of 30 m, and an overall accuracy higher than 90%, which can meet the needs of this study. Therefore, FROM-GLC is selected as the data source of ISA in this study.

As an important indicator of urban development process, impervious surfaces have attracted the attention of many scholars at home and abroad, and many studies have shown that a change of the total impervious surface area can effectively reflect the development and expansion of cities, and the spatial distribution characteristics of impervious surfaces are consistent with the built-up areas of cities, which can better reflect the level of urbanization [[12], [13], [14], [15], [16]]. For example, Yang et al. [17] in 2005 found that the spatial and temporal trends of their imperviousness index were consistent with the urban land use/cover changing trends obtained through image interpretation, and proposed that the urban imperviousness index could quickly and objectively assess urban spatial change characteristics. The study of impervious surface changes can be used to evaluate urban development trends; however, previous studies have focused on qualitative analyses of changes in spatial and temporal characteristics of cities, and quantitative analyses of impervious surfaces are relatively lacking. With the rapid development of remote sensing and spatiotemporal big data technology, the quantitative analysis of impervious surfaces has been promoted. Many scholars quantitatively analyze the characteristics of impervious surface changes using methods such as the ISA ratio, area growth amount and growth rate, urban expansion intensity index, etc. [[18], [19], [20], [21], [22], [23], [24]]. For example, Jian et al. [25] quantified the dynamic changes of impervious surfaces in Beijing by calculating the percentage of total ISA in 2001 and 2009. In studies of impervious surface driving forces, some other scholars [26] have used a regression analysis and boosted regression trees to analyze the drivers of impervious surface changes. Zhou et al. [27] analyzed the driving forces of spatial and temporal changes in the landscape pattern of impervious surfaces in Xiamen with a regression analysis. However, more previous studies only selected a few drivers which were then analyzed qualitatively. In summary, current research on urban impervious surfaces is mostly limited to the qualitative and quantitative analysis of spatial and temporal characteristics changes, and the research methods are typically the same, i.e., mostly calculating the expansion rate and dynamic degree to analyze the changes of impervious surfaces quantitatively. There is a lack of analyses regarding the changing trends of impervious surfaces. Studies on the factors affecting the spatial and temporal variations of impervious surfaces have basically been conducted using the data of the influencing factors in from a certain period, and the analyses considered the influence of individual factors on the spatial and temporal distribution characteristics of impervious surfaces.

Therefore, this paper takes the CPUA as the study area,obtains the spatial and temporal distribution characteristics of impervious surfaces in the CPUA in the T1 period (2000–2009) and the T2 period (2009–2018) with the help of the global artificial impervious surface dataset (GAIA); analyzes the trends of the area changes of the impervious surfaces of the CPUA from 2000 to 2018 using the Mann–Kendall (MK) trend test analysis method; uses the GeoDetector method to analyze the explanatory power of the amount of change of different influencing factors on the spatial and temporal changes of impervious surfaces in the CPUA in two periods. With these methods, we ascertain the dynamics of the amount of change of the influencing factors on the explanatory power of the spatial and temporal changes of impervious surfaces from period T1 to period T2. Finally, we use the interaction detection method to analyze the explanatory power of the interaction of two factors on the changes of impervious surfaces. The overall aim of this work is to provide scientific suggestions for the development planning and ecological construction of the CPUA, and provide some guidance for the spatial development strategies and the territorial spatial planning of other urban agglomerations in China.

## 2 Study area and data sources

### 2.1 Overview of the study area

**Fig. 1:**
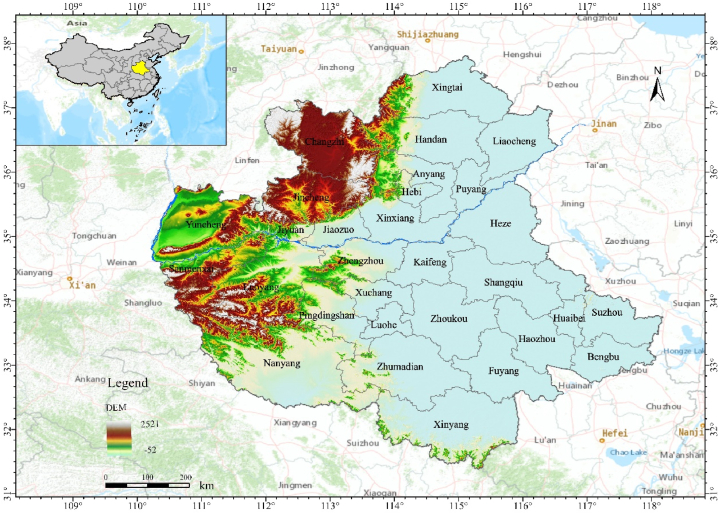
Study area.

The CPUA is located in the central-eastern part of China (see Fig. 1), bounded by latitudes 31°23′–37°47′N and longitudes 110°15′–118°10′E. It is dominated by Henan Province, covering 18 prefecture-level cities in Henan, three prefecture-level cities in Shanxi, Changzhi, Jincheng, and Yuncheng, two prefecture-level cities in Hebei, Handan, and Xingtai, two prefecture-level cities in Shandong, Liaocheng, and Heze, and five prefecture-level cities in Anhui, Suzhou, Huaibei, Fuyang, Haozhou, and Bengbu. The Central Plains region, with a total land area of 287,000 square kilometers, is the birthplace of the Chinese nation and Chinese civilization, and is currently a relatively large and densely populated urban agglomeration in China. At the end of 2017, the CPUA had a gross product of 677,812 million yuan, a total population of 188.8814 million people, and urbanization rate, i.e., the urban residential population reached 49.6% of the total population.

### 2.2 Data sources

The data used in this paper mainly include the impervious surface data of the CPUA, administrative division vector data, digital elevation model (DEM) image data, and socioeconomic data. The impervious surface data were obtained from the GAIA dataset published by Gong Peng's team at Tsinghua University [11], which can be downloaded from http://data.ess.tsinghua.edu.cn. The DEM data were obtained from the Geospatial Data Cloud and downloaded through https://www.gscloud.cn/. The socioeconomic data were obtained from the statistical yearbooks of Henan Province, Shanxi Province, Shandong Province, Anhui Province, and Hebei Province, including the gross domestic product (GDP) per capita, urbanization rate, greenery coverage area, health care, transportation, and the proportion of secondary and tertiary industries.

The GAIA dataset is in TIF format with a spatial resolution of 30 m. This dataset divides the globe into geographic grids. We downloaded the 63 images covering the study area, preprocessed the data based on the ArcGIS platform, performed mosaic processing on the 63 images, used the administrative boundary data of the CPUA to extract its mask, and obtained the CPUA from the GAIA data from 1985 to 2018.

## 3 Methods

### 3.1 Impervious surface growth dynamic degree

The growth rate of impervious surfaces can be expressed by the dynamic degree [28], where the single kinetic attitude is calculated by the following equation (1):

(1) $$D=\frac{A_{i}-A_{j}}{A_{j}}\times \frac{1}{T}\times 100\%\text{,}$$

where D is the dynamic degree, A_*i*_ is the total area of impervious surfaces in year *i*, A_*j*_ is the total area of impervious surfaces in year *j*, T is the length of time between year *i* and year *j*, and T is the number of years apart. The changes in the spatial and temporal characteristics of artificial impervious surfaces in the CPUA were studied by calculating the growth area and growth rate of impervious surfaces in two time periods from 2000 to 2009 and from 2009 to 2018. By calculating the total ISA and its spatial proportion, it can directly reflect the degree of impervious surface coverage in the CPUA, and calculating the growth rate of impervious surface can reflect the speed of urbanization process in the CPUA to a certain extent.

### 3.2 Factor change discretization processing

Since GeoDetector is good at analyzing type quantities, the data of each factor were discretized before using GeoDetector for our influencing factor analysis. The values before discretization for all factors except the DEM data were the absolute values of the difference between 2000–2009 and 2009–2018, obtained by the following equation (2):

(2) $$V_{i}=\left[A_{i}-B_{i}\right]\text{,}$$

where *V*_*i*_ is the difference between the *i*th factor in 2000 and 2009 or 2009 and 2018, *A*_*i*_ is the value of the *i*th factor in 2009 or 2018, and *B*_*i*_ is the value of the *i*th factor in 2000 or 2009.

The data of each influencing factor are discrete, including the DEM (F1), built-up area of greenery coverage (F2), GDP per capita (F3), transportation (F4), health care (F5), urbanization rate (F6), and the proportion of secondary and tertiary industries (F7). The greening coverage area of built-up areas refers to the ground covered by perennial vegetation within the built-up areas of cities and towns; in this work, the green belts on both sides of roads are the main statistical objects, in addition to squares, within companies and factories, within residential areas, etc., but excluding the vegetation replanted on the top floors of buildings. Transportation is expressed in terms of road mileage, while health care is expressed in terms of the number of health care institutions, including hospitals, health centers, sanatoriums, outpatient clinics, health clinics, and emergency stations. The urbanization rate is expressed as the ratio of urban population to the total population. The ratio of secondary and tertiary industries is the ratio of these industries to the GDP.

First, the statistical yearbook data of 2000, 2009, and 2018 were differenced to obtain the absolute values of the differences between 2009 and 2000 and 2018 and 2009. Then, the natural interruption points were classified according to the absolute value of the differences, that is, the degree of change of each factor data, and the classification results were assigned the values shown in Table 1 and Table 2(where the range of each factor is 1–7, where a larger number infers a higher degree of change among the factors, and vice versa).

**Table 1:** 2000–2009: discretization processing of the different factors.

value	F1	F2	F3	F4	F5	F6	F7
1	−182	406–514	2126–6444	1434–3581	11–54	≤5.11	0.08–2.45
2	130–321	515–809	6445–9199	3582–4200	55–144	5.12–11.58	2.46–4.74
3	321–564	810–1053	9200–10626	4201–6092	145–340	11.59–17.30	4.75–6.55
4	564–836	1054–1276	10627–13064	6093–7656	341–731	17.31–19.36	6.56–8.80
5	836–1108	1277–1652	13065–18086	7657–13767	732–1232	19.37–20.88	8.81–10.17
6	1108–1411	1653–2118	18087–20211	13768–17776	1233–1799	20.89–22.61	10.18–18.10
7	1411–2521	2119–7323	20212–32423	17777–29579	1780–4409	22.62–29.00	18.11–28.80

**Table 2:** 2009–2018: discretization processing of the different factors.

value	F1	F2	F3	F4	F5	F6	F7
1	−182	302–516	13510–16070	237–316	45–531	≤3.78	0.40–0.91
2	130–321	517–834	16071–20172	317–913	532–1480	3.79–7.91	0.92–2.11
3	321–564	835–1091	20173–23982	914–1153	1481–2531	7.92–10.00	2.12–3.57
4	564–836	1092–1484	23983–26789	1154–1881	2532–3504	10.01–10.90	3.58–5.21
5	836–1108	1485–2095	26790–30711	1882–2750	3505–4324	10.91–12.40	5.22–7.50
6	1108–1411	2096–4086	30712–37761	2751–3200	4325–6392	12.41–13.60	7.51–8.69
7	1411–2521	4087–10581	37762–57121	3201–6530	6393–9083	13.61–22.50	8.70–14.00

### 3.3 GeoDetector

GeoDetector is a new statistical method for detecting spatial differentiation and revealing the driving factors behind it. Developed by Wang et al. [29], it consists of four detectors: factor detection, interaction detection, risk detection, and ecological detection, where factor detection and interaction detection are used in this study.

Factor detection involves detecting the extent to which a factor, that is, the independent variable, X, influences the spatial variance of the dependent variable, Y. The magnitude of the *q*-value [30] is used to measure the degree of X's influence. The *q*-value takes a range from 0 to 1, where the closer the *q*-value is to 1, the greater the degree of X's influence on Y, and vice versa. In special cases, a *q*-value of 1 indicates that X completely determines the spatial distribution of Y, and a *q*-value of 0 indicates that X has nothing to do with Y. In summary, the *q*-value indicates that the factor X determines 100 × *q*% of Y, which is expressed as equation (3):

(3) $$q=1-\frac{\sum_{h=1}^{L}N_{h}\sigma_{h}^{2}}{N\sigma^{2}}\text{,}$$

where *h* is the classification of variable Y or factor X, N_*h*_ and N are the number of cells of type *h* and the whole area, respectively, and σ_*h*_^2^ and σ^2^ are the variances of the Y values of type *h* and the whole area, respectively.

Interaction detection detects whether the degree of influence on the dependent variable Y is strengthened or weakened when two factors act together, and whether the two factors are independent of each other in their influence on Y. The types of interaction between two factors are nonlinear weakening, single-factor nonlinear weakening, two-factor enhancement (TFE), independent of each other, and nonlinear enhancement (NE).

### 3.4 The MK test

#### 3.4.1 MK trend test

To test the trends of the impervious surface changes, the MK trend test method is used here to test the year-to-year incremental changes of the impervious surfaces, which assumes that the time data series is an independent, random, homogeneously distributed sample series. For time series X, the MK trend test statistic *S* is expressed as equation (4):

(4) $$S=\sum_{i=2}^{n}\sum_{j=1}^{i-1}sgn\left(x_{i}-x_{j}\right)\text{,}$$

where *x*_*i*_ is the *i*th data value of the time series and *n* is the length of the data sample. *sgn* is the sign function, which is defined by the following equation (5):

(5) $$sgn\left(\theta \right)=\left\{ \begin{array}{cc} 1 & \left(\theta >0\right)\\ 0 & \left(\theta =0\right)\\ -1 & \left(\theta <0\right) \end{array}\right.\text{,}$$

*S* is a normal distribution with a mean of 0 and its variance is given below in equation (6):

(6) $$Var\left(S\right)=\frac{n\left(n-1\right)\left(2n+5\right)-\sum_{i=1}^{n}t_{i}\left(i-1\right)\left(2i+5\right)}{18}\text{,}$$

*Z* is the standardized statistic and is calculated by the following equation (7):

(7) $$Z=\left\{ \begin{array}{cc} \left(S-1\right)/\sqrt{Var\left(S\right)} & S>0\\ 0 & S=0\\ \left(S+1\right)/\sqrt{Var\left(S\right)} & S<0 \end{array}\right.\text{,}$$

where Z is the test value, and if Z is greater than 0, the test time series has an upward trend, and if Z is less than 0, the test time series has a downward trend. Absolute values of *Z* greater than or equal to 2.32, 1.64, and 1.28 indicate that they pass the significance test at 99%, 95%, and 90% confidence levels, respectively.

#### 3.4.2 MK mutation test

First, for a time series, X, containing *n* samples, we construct an order column as equations (8), (9):

(8) $$s_{k}=\sum_{i=1}^{k}r_{i}\;(k=2,3,\cdots ,n)\text{,}$$

where:

(9) $$r_{i}=\left\{ \begin{array}{cc} +1 & x_{i}>x_{j}\\ 0 & otherwise \end{array}\right.\;\left(j=1,2,\cdots ,i\right)\text{,}$$

The order column *s*_*k*_ is the accumulation of the number of values at the *i*th moment when the value is greater than the *j*th moment.

Under the assumption that the time series is random, we define the following statistic in equation (10):

(10) $$UF_{K}=\frac{\left[s_{k}-E\left(s_{k}\right)\right]}{\sqrt{Var\left(s_{k}\right)}}\;\left(k=1,2,\cdots ,n\right)\text{,}$$

where *UF*_*1*_ = 0, *E(s*_*k*_*)* and Var*(s*_*k*_*)* are the mean and variance of *s*_*k*_, respectively, and *x*_*1*_, *x*_*2*_, …,*x*_*n*_ have the same continuous distribution when they are independent of each other, which can be deduced from the following equations (11), (12):

(11) $$E\left(s_{k}\right)=\frac{k\left(k-1\right)}{4}\;\left(2\leq k\leq n\right)\text{,}$$

(12) $$Var\left(s_{k}\right)=\frac{k\left(k-1\right)\left(2k+5\right)}{72}\;\left(2\leq k\leq n\right)\text{,}$$

where UF_*k*_ is a standard normal distribution, which is a sequence of statistics calculated in the order of time series X (x_1_, x_2_, …,x_n_), given a significance level α. If UF_*i*_ > U_α_, it indicates that there is a significant trend change in the series. The above procedure is repeated in the inverse order of the time series X (x_n_, x_n-1_, …,x_1_), where we let UB_*k*_=UF_*k*_(*k* = *n*, *n*-1, …,1), UB_1_ = 0. The obtained statistical series are UF_*k*_ and UB_*k*_, where the positive and negative values of UF_*k*_ indicate the trend of x_*k*_; here, a positive value indicates an upward trend of the series, and a negative value indicates a downward trend. When UF_*k*_ and UB_*k*_ exceed the critical line of significance, it indicates a significant trend. Finally, the UB_*k*_ and UF_*k*_ curves are plotted, and if there are intersections of these two curves in the confidence interval |U| ≤ 1.96, then these intersections are the mutation points of this sequential order.

## 4 Results and discussion

### 4.1 GAIA from 2000 to 2018

**Fig. 2:**
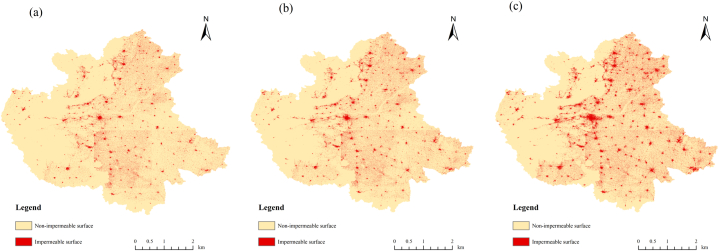
Spatial distribution of the ISAs in the CPUA in 2000(a), 2009(b) and 2018(c).

The GAIA dataset values range from 0 to 34, which indicate different years, with 0 indicating non-impermeable surfaces and values greater than 0 indicating impermeable surfaces. For the study of this paper, three years of data from 2000, 2009, and 2018 were selected, so the GAIA data images of the CPUA from 1985 to 2018 had to be extracted to obtain finally the artificial impervious surface (GAIA) data of the CPUA in 2000, 2009, and 2018 (see Fig. 2).

**Fig. 3:**
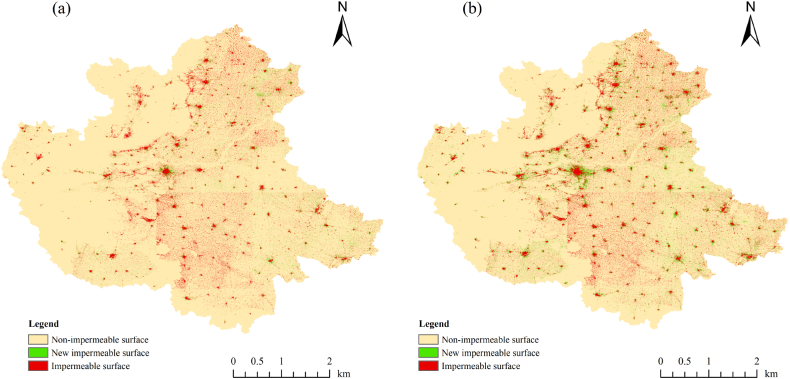
Spatial changes of the ISAs in the CPUA, 2000–2009(a) and 2009–2018(b).

The extracted images of 2000 and 2009 and 2009 and 2018 were subjected to map algebra to obtain the spatial variation maps of impervious surfaces for the two time periods of 2000–2009 (T1) and 2009–2018 (T2) (see Fig. 3). Then, the spatial and temporal variation characteristics of impervious surfaces in the two time periods in the CPUA were analyzed.

From the spatial distribution map and change distribution map of impervious surfaces in the CPUA, we can see that the impervious surfaces of the CPUA are consistent with the morphology of urban built-up areas. This is consistent with the urban boundary data produced by Li et al. in terms of the spatial morphological distribution [31], which shows the distribution pattern of “one main multi-point”; that is, the central urban area is the main area, and the surrounding counties and industrial parks and industrial zones are the points, and the changes of impervious surfaces are centered on each point, showing radial expansion. On the whole, the expansion of impervious surfaces in the CPUA mainly occurs in the eastern region. It is obvious from the figure that from 2009 to 2018 (T2 period), the impervious surfaces of the CPUA increased much faster than from 2000 to 2009 (T1 period). Over the whole study period, the trend of transformation to impervious surfaces around the urban centers became increasingly obvious, and the urban expansion became obviously enhanced.

This may be influenced to a large extent by the economy, infrastructure, population migration and intra-city linkages, In 2006, the “Central Plains City Cluster Overall Development Plan Outline” was promulgated. The CPUA Development Plan has entered the implementation phase, which has somewhat accelerated the urbanization of the CPUA. The CPUA, in the midst of rapid development, is affected by the level of economic development and the construction of infrastructure within the cities, which have insufficient population and resource carrying capacity and an increased demand for land. As an important link between cities, transportation is also growing rapidly, which in turn is contributing to the rapid increase in the amount of impervious surfaces.

### 4.2 Analysis of the dynamic degree and trends of ISA changes

**Fig. 4:**
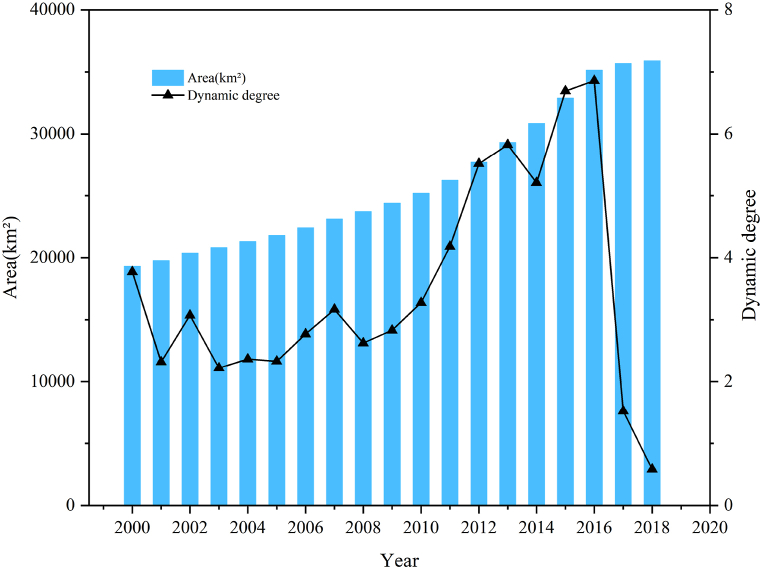
ISA statistics and year-by-year dynamic degree from 2000 to 2018.

Based on the ArcGIS platform, we extracted the ISA data year-by-year from 2000 to 2018 and statistically analyzed the area data. It can be seen from Fig. 4 that the ISA of the CPUA increased from 19,304.01 km^2^ in 2000 to 35,887.16 km^2^ in 2018, among which the T1 and T2 periods increased by 5083.78 km^2^ and 11,499.36 km^2^, respectively. The percentage of impervious surfaces increased from 5.55% in 2000 to 10.33% in 2018, among which the T1 and T2 periods increased by 1.46% and 3.31%, respectively.

Further calculating the year-by-year dynamic degree of impervious surfaces from 2000 to 2018, as shown in Fig. 4, the dynamic degree of impervious surfaces was 2.93% and 5.24% in the T1 and T2 periods, respectively. It can be seen that the growth rate of impervious surfaces in the CPUA in the T2 period before 2017 was much higher than that in the T1 period, where the growth amount reached more than twice of that in the T1 period, and overall, the year-by-year dynamic degree in the T2 period was greater than that in the T1 period.

**Fig. 5:**
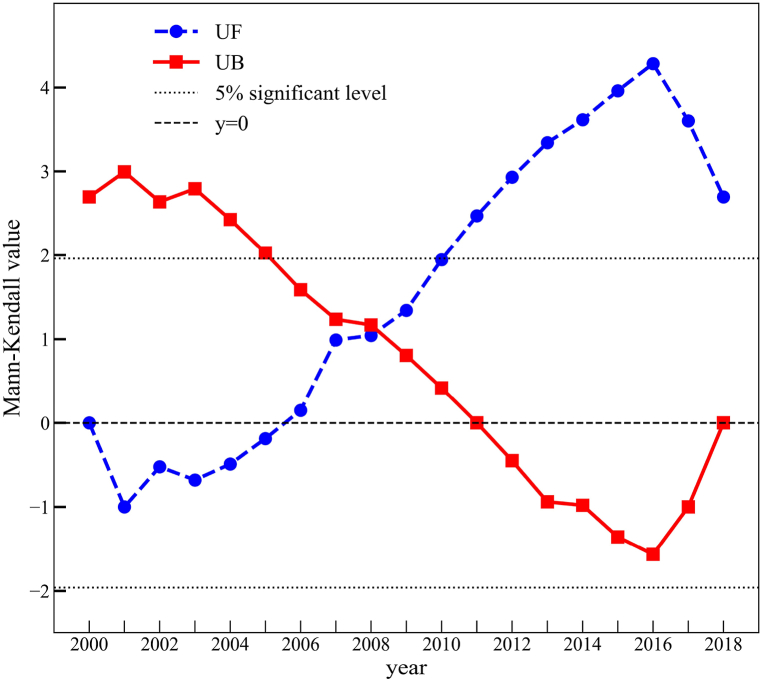
MK statistical curve of the ISA changes from 2000 to 2018.

In order to study the characteristics and trends of changes in the ISA of the CPUA, a MK test trend analysis and mutation test were used, and it was calculated that the statistical variable S value of the calculated test was 77, the standardized statistic Z-value was 2.6589, and the P-value was 0.0078. At a confidence level of 0.05, the trend test value of 2.6589 > 1.96 (critical value) indicates that the interannual increment of the ISA of the CPUA passed the significance trend test at the 95% confidence level; that is, the interannual increment of the ISA of the CPUA showed an increasing trend and exhibited significance characteristics. The UF and UB curves were calculated by the year-to-year change in the ISA during 2000–2018 (see Fig. 5). From the UF curve, it can be seen that after 2005, its value is greater than 0, and the ISA changes show an upward trend, and after 2010, it is greater than the value of the critical straight line, which indicates a significant upward trend. Further observing the UF and UB curves, it is found that their intersection point is in 2008, which is located between the critical values, indicating that in 2008 the interannual increment of the ISA showed a sudden change in the state of growth.

Overall, the year-by-year dynamic degree of the T2 period is greater than that of the T1 period, which is probably due to the accelerated urbanization during the T2 period, prompting the transformation of permeable surfaces to impermeable surfaces. During the T2 period, the strong rise of the core cities through initiatives such as the strong provincial capital strategy led to high economic growth, which was accompanied by a curtailment of the population outflow and an acceleration of urbanization. Specifically, the dynamic degree of impervious surfaces showed fluctuating slow growth at first and rapid growth after 2007. Initially, urbanization was affected by the limited radiation-driven capacity of the central city, an inadequate coordination mechanism, and the weak radiation-driven role of the core city, thus limiting urban expansion to a certain extent. After 2007, the accelerated implementation of the Central Rising Strategy and the economic stimulus package has led to a significant change in the drivers of urbanization. Cities expanded rapidly during this phase, resulting in a rapid expansion of impervious surfaces and a large increase in impervious surface coverage [32], which may be an important reason for the rapid growth of the dynamic degree after 2007 and the sudden growth of ISA in 2008.

After 2016, the interannual incremental changes of the impervious surfaces in the CPUA and the dynamic degreehave both decreased. The reason for this is because the overall land utilization rate of the CPUA showed an upward trend from 2005 to 2016 and reached the highest value in 2016 [33]. An improved land use efficiency can improve the economic, social, and environmental benefits per unit of land area, reduce the increase of the total ISA, while ensuring the realization of high-quality socio-economic development, helping realize the concurrent and intensive development of the city.

### 4.3 Spatial and temporal change distribution characteristics of the influencing factors

**Fig. 6:**
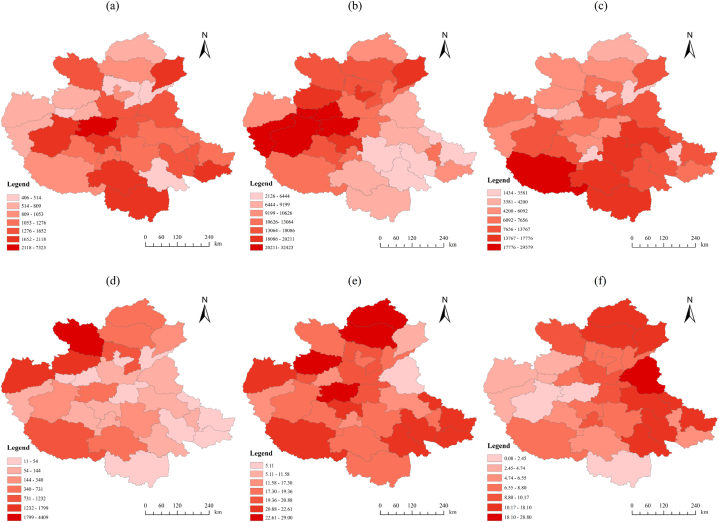
Grading chart of influencing factor changes from 2000 to 2009, built-up area of greenery coverage (a), GDP per capita (b), transportation (c), health care (d), urbanization rate (e), and the proportion of secondary and tertiary industries (f).

Based on the ArcGIS platform, used to display the change amount of each influencing factor in a hierarchical manner and visualize each influencing factor, according to the obtained hierarchical map of the change of influencing factors in each city of the CPUA, we can see that in the T1 period (see Fig. 6), the largest change in F2 was in Zhengzhou, followed by Xuchang and Xinyang, and the smallest changes were in Fuyang and Anyang. The largest changes in F3 were in Zhengzhou and Jiyuan, and the smallest change was in Zhoukou. The most significant change in F4 was in Nanyang, and the smallest changes were in Shangqiu and Luohe. The largest change in F5 situation was in Changzhi, and the smallest changes were in Bengbu and Jiyuan. The more significant changes in F6 were in Jincheng, Handan, and Zhengzhou, and the smallest change was in Heze. The largest change in F7 was in Heze, and the smallest change was in Luoyang.

**Fig. 7:**
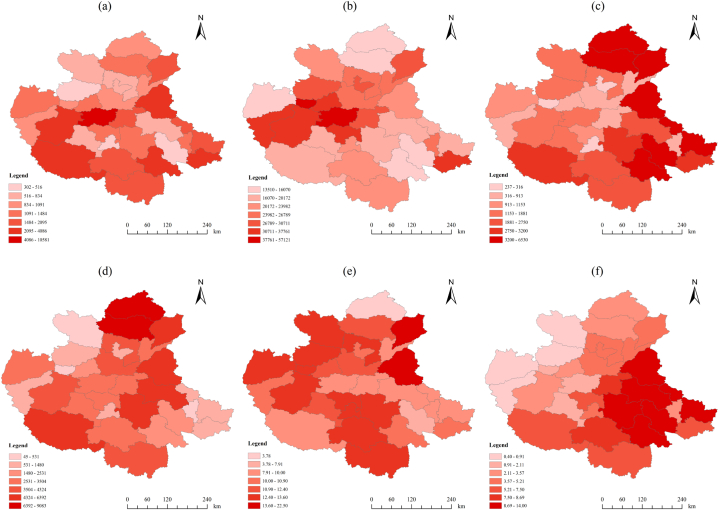
Grading chart of influencing factor changes from 2009 to 2018, built-up area of greenery coverage (a), GDP per capita (b), transportation (c), health care (d), urbanization rate (e), and the proportion of secondary and tertiary industries (f).

In the T2 period (see Fig. 7), F2 had the largest change in Zhengzhou, followed by Heze and Luoyang, and the smallest changes were in Luohe and Haozhou. F3 had the largest changes in Zhengzhou and Jiyuan, and the smallest change in Handan. F4 had the largest changes in Fuyang and Handan, and the smallest changes in Luohe and Hebi. F5 had the most significant institutional changes in Handan and Xingtai, and the smallest change in Changzhi. F6 had the most significant changes in Heze and Liaocheng, and the smallest change in Xingtai. F7 had the largest changes in Suizhou and Zhoukou, and the smallest changes in Jincheng and Sanmenxia.

Further, the Arcgis 10.2 and GeoDa software were used to calculate the Moran index of the influencing factor distributions, analyze the global spatial autocorrelation characteristics of the influencing factors in the T1 and T2 periods, and test whether there is an aggregation of influencing factors in the CPUA; the calculation results are shown in Table 3.

**Table 3:** Moran index (I-value) of the influencing factor distributions.

	F2	F3	F4	F5	F6	F7
T1 period	0.03	0.51	0.19	0.16	0.04	0.21
T2 period	−0.18	0.27	0.21	0.27	0.01	0.49

When the Moran index is greater than 0, it means that the influencing factor presents a positive spatial correlation, where the larger its value, the more obvious the spatial correlation; when the Moran index is less than 0, it means that the influencing factor presents a negative spatial correlation, where the smaller its value, the greater the spatial difference. The calculation results show that F3 is at a 0.01 significance level and was significant in the T1 period, and the Z-value is greater than the critical value of 2.58, which indicates that F3 has a 99% certainty of producing an agglomeration effect in its spatial distribution; that is, F3 in the CPUA shows the aggregation characteristics of a high-high value and a low-low value in spatial distribution. The P-values of F4, F5, and F7 are less than 0.1, implying confidence levels of 90%, and their Z-values are greater than the critical value of 1.65, indicating that F4, F5, and F7 have a 90% certainty of producing a clustering effect in their spatial distributions. The P-values of F2 and F6 are greater than 0.1, indicating that their clustering effect is not significant. In the T2 period, the P-value of F7 is less than the 0.01 significant level, i.e. its confidence level reaches 99%, and its Z-value score is greater than the critical value of 2.58, indicating that F7 has a 99% certainty of producing an agglomeration effect in its spatial distribution; that is, F7 of the CPUA shows the aggregation characteristics of a high-high value and a low-low value in space. The P-values of F3 and F5 are less than the 0.05 significance level, i.e., they have a 95% confidence level, and their Z-value scores are greater than the critical value of 1.96, indicating that F3 and F5 have a 95% certainty of producing an agglomeration effect in their spatial distributions. The P-value of F4 is less than 0.1, implying a confidence level of 90%, and its Z-value is greater than the critical value of 1.65, indicating that F4 has a 90% certainty of producing an agglomeration effect in its spatial distribution. The P-value of F6 is greater than 0.1, indicating that its agglomeration effect is not significant. In particular, the Moran index of F2 is less than 0, indicating that it shows a negative correlation in space, which is manifested as heterogeneous clustering, that is, high and low values of agglomeration. The I- and Z-values of F3 in period T2 decrease compared to period T1, indicating that the economy of the CPUA tends to develop in an overall balanced manner and the spatial agglomeration effect is weakened.

**Fig. 8:**
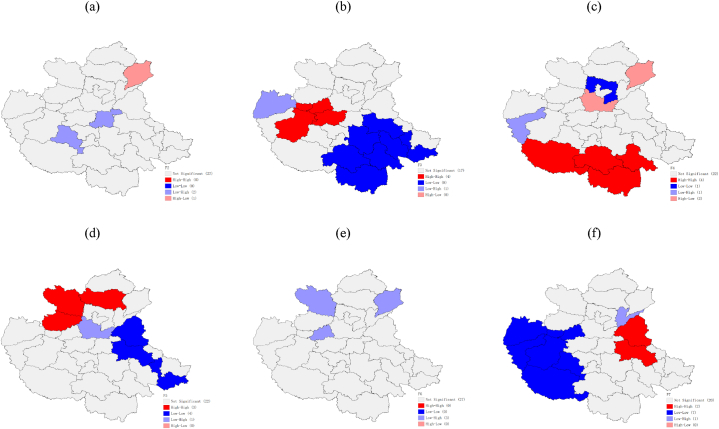
Local autocorrelation LISA aggregation plot of the amount of change in impact factors from 2000 to 2009, built-up area of greenery coverage (a), GDP per capita (b), transportation (c), health care (d), urbanization rate (e), and the proportion of secondary and tertiary industries (f).

**Fig. 9:**
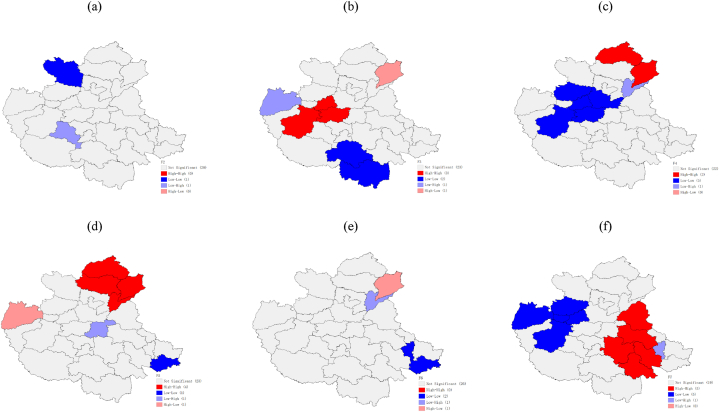
Local autocorrelation LISA aggregation plot of the amount of change in impact factors from 2009 to 2018, built-up area of greenery coverage (a), GDP per capita (b), transportation (c), health care (d), urbanization rate (e), and the proportion of secondary and tertiary industries (f).

The local autocorrelation characteristics of the amount of variation of the impact factors were analyzed using the Geoda software, and the local LISA aggregation plots shown in Fig. 8 and Fig. 9 were obtained. From the figures, it can be seen that the overall spatial correlation of the amount of green cover change in built-up areas in the CPUA during 2000–2018 is not significant, with a few cities showing a negative autocorrelation, and only Liaocheng is surrounded by low values while Kaifeng and Pingdingshan are surrounded by high values during the T1 period. By the T2 period, Pingdingshan is still surrounded by high values, while Changzhi shows a low value aggregation feature, which may be the reason for the negative global Moran index. It shows that Kaifeng may have started to pay attention to urban green development driven by the surrounding areas, while for Pingdingshan, there is still need to strengthen its urban greening construction. The spatially positive autocorrelation of GDP changes in the CPUA during 2000–2018, with strong “high-high” agglomeration characteristics in Zhengzhou, Jiaozuo and Luoyang, and strong “low-low” agglomeration characteristics near Xinyang and Zhumadian in the southeast, are basically consistent with the findings of Zhao et al. [34]. In the T2 period, the cities in the high-value areas still maintain their advantages, while the per capita GDP in the low-value areas grows significantly, and the “low-low” characteristic of Zhoukou, Fuyang, and Huzhou disappears, indicating that in the economic development of the CPUA, Zhengzhou City, as the core city, has had a radiating effect on its surrounding cities [34]; meanwhile, Xinyang City, the southernmost city, remains low because it is far away from the central city and has difficulty in being influenced by the central city. The amount of change in the number of road miles in the CPUA has a clear clustering. In the T1 period, “low-low” clustering is seen in Anyang, “high-high” clustering in Zhumadian, Nanyang, Fuyang and Xinyang in the south, “high-high” clustering in Xingtai and Liaocheng, and “low-low” clustering in Zhengzhou, Jiaozuo, Luoyang, Xinxiang, and Jincheng in the T2 period. These indicate that the transportation within the CPUA is relatively well developed and has less development potential, while the external urban transportation system is being built faster and the number of road miles is increasing more.

From the local LISA clustering picture of the change in the number of medical institutions, it can be seen that during the T1 period, Handan, Changzhi, and Jincheng in the north show “high-high” clustering characteristics, while Heze, Shangqiu, Huaibei, and Bengbu in the east show “low-low” clustering characteristics. In the T2 period, Xingtai, Handan, Puyang, and Liaocheng in the north show “high-high” agglomeration characteristics, while only Bengbu city is located in the low-value areas, indicating that the cities located in the high-value areas played a leading role and influenced the medical development of the surrounding cities, and the low-value agglomeration area was obviously reduced. From the local LISA agglomeration picture of urbanization rate change, we can see that in period T1, three cities, Changzhi, Liaocheng, and Jiaozuo, are in low-value areas surrounded by high-value areas. In period T2, only Puyang is surrounded by high-value areas, and the global Moran index is close to 0 in both periods, indicating that the agglomeration of the urbanization rate change is not significant and the overall spatial distribution is random. The proportion of secondary and tertiary industries in the CPUA has obvious clustering characteristics. In the T1 period, Heze and Shangqiu showed “high-high” aggregation characteristics, while seven cities in the west, such as Yuncheng and Sanmenxia, showed “low-low” aggregation characteristics. In the T2 period, the industrial development of three cities in the west, such as Nanyang, Sanmenxia and Pingdingshan, accelerated, and the “low-low” aggregation characteristics disappeared. The eastern cities maintained their industrial advantages and played a leading role in industry, pulling the industries of the Zhoukou, Huzhou, and Fuyang cities to the south, so the five southeastern cities show “high-high” gathering characteristics.

### 4.4 Influencing factor detection

**Fig. 10:**
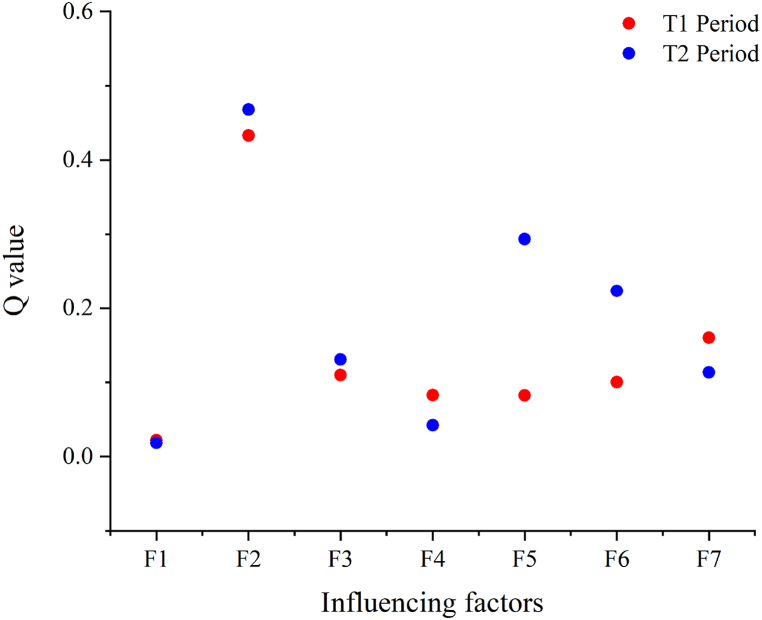
Scatter plot of the explanatory power of the impact factor *q*-values.

The discrete data of the spatial and temporal variations of each influencing factor and the dependent variable data were factored using GeoDetector. The calculated *q*-values of each factor are shown in Table 4, and the scatter distribution of *q*-values is shown in Fig. 10.

**Table 4:** Influencing factor detection results.

	F1	F2	F3	F4	F5	F6	F7
T1 period	0.0221	0.4331	0.1099	0.0826	0.0822	0.1003	0.1601
T2 period	0.0187	0.4678	0.1307	0.0423	0.2934	0.2236	0.1132

The results of the single-factor detection show that the influencing factor F2 has the strongest explanatory power on the change of the ISA in the T1 and T2 periods, and F1 has insignificant explanatory power. The small change in the *q*-value of F1 indicates that the small effect of the change in the DEM on the spatial divergence of ISA changes during urban expansion is due to the fact that the DEM hardly changes in a short period. The construction of urban greening facilities is included in the impervious surface construction. In the process of urban expansion, the change of greening coverage area, to a certain extent, reflects the level of urban construction expansion and urban ecological construction. The changes in green coverage area in the built-up areas have the strongest explanatory power for the changes in the ISA, indicating that the changes in green coverage area and ISA have strong similarity in terms of spatial distributions, and the urban construction in the CPUA is integrated with ecological development, focusing on the construction of ecological cities [35]. The *q*-value of F2 slightly increased in the period of T2. On the one hand, the increase of urban construction land is an unavoidable process, which is coupled with people's increasing requirements for living environments and the guidance of relevant policies; hence, more attention is paid to environmental greening than urban construction. On the other hand, the green development levels of the cities in the CPUA show an overall increasing trend, and urban expansion and ecological construction have strong consistency. This shows that the urban expansion process in the CPUA does not neglect urban greening construction because of urban expansion, but pays more attention to the connotative development and the development of urban green space.

The higher increase in per capita GDP indicates a faster urban economic development and a greater demand for construction land, which in turn increases the area of impervious surfaces. As such,the explanatory power of F3 increases in the T2 period, probably because the growth rate of per capita GDP in the CPUA is greater than the expansion rate of ISA, and urban spatial expansion lags behind economic development. Thus, cities with good traffic conditions can form competitive location advantages [36], and traffic development to a certain extent promotes the rapid expansion of urban lands [37,38], and the planning and construction of the Central Plains City Cluster “meter” type high-speed railway has promoted the rapid expansion of urban construction land. The explanatory power of both the T1 and T2 periods is small, which also indicates that the region's traffic has reached a certain scale and is more mature, and its incremental spillover effect on urban spatial growth will further diminish. The increase in health care facilities is indicative of an indicator related to the increase in urban construction land area. Earlier, there was a lack of appropriate policies and strong enforcement for the construction of public infrastructure (health care). During the T2 period, the state invested more in public infrastructure and formulated a series of policies for urban and rural areas, i.e., to improve the public health care system further in urban areas and build a health care system with comprehensive coverage in rural areas; concurrently, relevant policies were effectively implemented during this period, and its *q*-value increased significantly.

The urbanization rate indicator used in this paper refers to population urbanization, which is an important indicator reflecting the urbanization process, and the population factor is an important influencing factor driving urban land expansion [39,40]; i.e., population growth is a direct driver of urban expansion. As the population grows, the demands for housing, education, and healthcare increase, bringing with them the constant expansion of cities and an increase in ISA size. A small change of the population urbanization rate in the early period is not enough to drive an increase of ISA in peri-urban areas, which may be the reason why the *q*-value in T1 period is smaller than that in the T2 period, indicating that there is a certain difference between population urbanization and spatial urbanization, and the coordination of population growth and land use growth should be further improved [41]. The development of secondary and tertiary industries also has a role to play in urban land use change. The continuous development of industries will lead to the extension of industries to the suburbs and sub-developed cities, promoting expansion of the urban scale [42]. In the later period, due to the implementation of the rural revitalization strategy, the investment in agricultural facilities has increased and the development of the primary industry has largely contributed to the increase in the amount of impervious surfaces of the countryside, making the explanatory power of the secondary and tertiary industries relatively lower.

### 4.5 Detection factor interactions

**Fig. 11:**
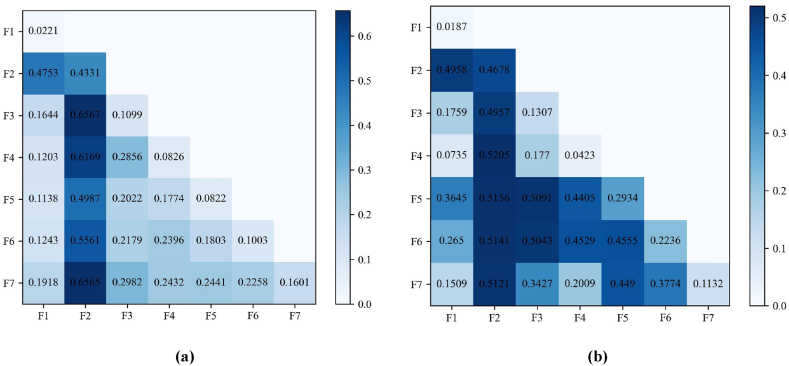
*q*-values for interaction detection of impact factors in periods T1(a) and T2(b).

The two-by-two interaction detections of each factor using the GeoDetector are shown in Table 5 and Fig. 11. The *q*-values of the interaction detection results among the seven influencing factors are greater than the *q*-values of the explanatory power obtained from a single factor, and the interaction of any two factors shows two results, i.e., NE or TFE, indicating that the factors are not independent of each other. The changes of ISA in the CPUA are influenced by the joint effect between seven influencing factors, and the interaction between two factors can have more explanatory power than a single factor on the spatial variation of ISA changes, where the interaction of two factors A and B satisfies q(A)+q(B)>q(A∩B)>max[q(A), q(B)] for TFE and q(A)+q(B)<q(A∩B) for NE.

**Table 5:** Influence factor interaction detection results.

T1 Period	F1	F2	F3	F4	F5	F6
F2	NE					
F3	NE	NE				
F4	TFE	NE	NE			
F5	NE	TFE	NE	NE		
F6	NE	NE	NE	NE	TFE	
F7	NE	NE	NE	NE	NE	TFE
T2 Period	F1	F2	F3	F4	F5	F6
F2	NE					
F3	NE	TFE				
F4	NE	NE	NE			
F5	NE	TFE	NE	NE		
F6	NE	TFE	NE	NE	TFE	
F7	NE	TFE	NE	TFE	NE	NE

In the T1 period, the stronger interaction for ISA changes was the combination of F2 with each other factor, and the top three factor combinations in terms of explanatory power *q*-value were F2 ∩ F3 (0.6567), F2 ∩ F7 (0.6565), and F2 ∩ F4 (0.6169), and the top three factor combinations in terms of interaction in the T2 period were still closely related to F2, which was in good agreement with the factor detection results. From the heat map of the interaction results, we can see that the *q*-value of the interaction results in the T2 period is larger, especially the interaction between F2 and F3 and other factors is more obvious, and we should continue to strengthen the urban greening construction and promote the high-quality economic development of the CPUA in the future, in order to utilize better their interaction with other factors and jointly promote the healthy and stable urban development of the CPUA in the process of urbanization.

Overall, the results of the interactions of F3 with other factors besides itself are NE, indicating that the change in GDP per capita and transportation development not only have some explanatory power on their own for the change in ISA in the CPUA, but also have enhanced explanatory power when interacting with other factors and exceed the sum of the effects of the two single factors. The level of economic development of the city and the investment in greening are the main factors affecting the greening coverage, and the economy provides a strong guarantee for the construction of urban green space [43]. As the economy grows, many factories and industrial areas are formed, which increase the demand for labor and employment opportunities, promoting population migration [39]. Regional economic growth is closely related to urban industrial clusters, and industrial agglomerations can promote regional economic development. The development of transportation and the construction of high-speed railroads and intercity railroads have obvious promotion effects on the economic development of the CPUA, and also on the industrial structure adjustment and upgrading of the CPUA. Economic prosperity and development provide a source of funding for medical construction that can support the construction and development of medical institutions, which may account for the increased explanatory power of F3 interacting with the other factors for ISA changes in the CPUA. By improving urban transportation, HSR promotes population mobility, increases employment opportunities, and boosts population growth in cities along the route [36], which may be the reason for the more prominent performance of F4 in the interaction. By improving urban transportation, HSR promotes population mobility, increases employment opportunities, and boosts population growth in cities along the route, which may be the reason for the more prominent performance of F4 in the interaction. In particular, the single factor influence of F4 was only 0.0423 in the T2 period, but the explanatory power of the factors exceeded 0.4 after interacting with F2, F5 and F6, indicating that F4 constituted a synergistic enhancement effect with F2, F5 and F6, which together explained the spatially divergent characteristics of the impervious surfaces in that period.

The basic principle of geographic detectors is that if the independent variable has a strong explanatory power for the dependent variable, then they also has some similarity in their spatial distributions [44]. In this paper, the factor detection and interaction detection methods of GeoDetector are used to explore the explanatory power of the changes of different factors on the changes of ISA in the CPUA and the interaction between two factors, which makes up for the shortcomings of the conventional methods that cannot explain the influence mechanism of the interaction [30,45],and it has some practical significance for a comprehensive understanding of the process and mechanism of ISA changes in the CPUA. It also has some practical significance for a comprehensive understanding of the process and mechanism of ISA changes in the CPUA. In contrast, a correlation analysis only considers linear relationships between variables, while ignoring other possible nonlinear relationships and complex interactions, and in addition, there may be unobserved hidden or confounding variables that may affect the correlation between variables. In the process of using the GeoDetector method, the discretization method used for the independent variables and the number of sampling points may have an impact on the results, and these limitations remain to be further investigated.

## 5 Conclusions and prospects

In this paper, using the CPUA as the study area and based on a GAIA dataset, the spatial and temporal variation characteristics and influencing factors of the impervious surfaces of the CPUA were studied using an MK trend test and GeoDetector, and it was found that.

(1) The impervious surfaces of the CPUA expanded significantly outward from 2000 to 2018, and the total ISA in 2018 was 1.86 times larger than in 2000. From 2000 to 2009, the total ISA expanded not only in the original basis of each urban center, but also more in the suburbs to form new points for expansion. In 2009–2018, the impervious surfaces mainly manifested near and around their origins. In general, the impervious surfaces of the CPUA showed outward expansion from each city center, and the development of the surrounding areas was driven by the priority development of the city centers; further development of the CPUA is guided by the policies related to the Central Plains region. The results of the trend analysis via the MK tests show that the interannual increment of the ISA in the CPUA is on an upward trend and shows a significant feature, that is, the impervious surfaces of the CPUA are undergoing a continuous outward expansion.
(2) From 2000 to 2018, the influencing factor F2 had the strongest explanatory power for the ISA changes in the CPUA, while the explanatory power of F1 was not significant. In the T2 period, the ISA changes in the CPUA changed from industrial transportation-driven to population-driven. In terms of influencing factor interaction, the influencing factors were enhanced by TFE and NE. The interaction between factors on the explanatory power of ISA changes in the CPUA is always greater than that of individual factors. It is important to pay more attention to urban greening in urban planning and design, so as to improve urban air quality, hydrological cycle and ecosystem services, reduce water quality pollution, and improve the residents' quality of life, all of which are important for creating a livable, healthy, and sustainable urban environment.

There are certain shortcomings in this study: the analysis of the GAIA data and influencing factor data are not sufficient, where only the data of two periods were compared. The socioeconomic influencing factors of the CPUA were not completely considered, which may make the analysis results of individual factors contingent, and the selection and analysis of the influencing factors still need to be improved. In a follow-up study, we will use other impervious surface data for a comparative analysis, select more comprehensive influencing factors, select data from multiple periods for a comparative study, explain the change patterns of the impervious surfaces in the CPUA from more aspects, analyze the driving mechanism of impervious surface change, further study the ecological and environmental effects of impervious surfaces, and provide some scientific basis for the internal development and sustainable development of the CPUA.

## Author contributions statement

Chunhong Zhao: Conceived and designed the experiments, Performed the experiments, Analyzed and interpreted the data, Wrote the paper. Huabo Zhang: Performed the experiments, Analyzed and interpreted the data, Wrote the paper. Haiying Wang: Conceived and designed the experiments, Analyzed and interpreted the data, Wrote the paper. Jinyi Zhao: Analyzed and interpreted the data, Wrote the paper.

## Data availability statement

The authors do not have permission to share data.

## Declaration of competing interest

The authors declare that they have no known competing financial interests or personal relationships that could have appeared to influence the work reported in this paper.
